# Diffusible signal factors (DSFs) bind and repress VirF, the leading virulence activator of *Shigella flexneri*

**DOI:** 10.1038/s41598-023-40023-w

**Published:** 2023-08-14

**Authors:** Rita Trirocco, Martina Pasqua, Angela Tramonti, Bianca Colonna, Alessandro Paiardini, Gianni Prosseda

**Affiliations:** 1 Institute Pasteur Italia, Department of Biology and Biotechnologies “Charles Darwin”, Sapienza University of Rome, p.le Aldo Moro 5, 00185 Rome, Italy; 2 Institute of Molecular Biology and Pathology, National Research Council, Rome, Italy; 3 Department of Biochemical Sciences, Sapienza University of Rome, p.le Aldo Moro 5, 00185 Rome, Italy

**Keywords:** Bacteriology, Pathogens

## Abstract

*Shigella*, the aetiological agent of human bacillary dysentery, controls the expression of its virulence determinants through an environmentally stimulated cascade of transcriptional activators. VirF is the leading activator and is essential for proper virulence expression. In this work, we report on in vitro and in vivo experiments showing that two autoinducers of the DSF family, XcDSF and BDSF interact with the jelly roll module of VirF causing its inhibition and affecting the expression of the entire virulence system of *Shigella*, including its ability to invade epithelial cells. We propose a molecular model explaining how the binding of XcDSF and BDSF causes inhibition of VirF by preventing its dimerization. Overall, our experimental results suggest that XcDSF and BDSF may contribute to ”colonisation resistance” in the human gut or, alternatively, may be exploited for the fine-tuning of *Shigella* virulence expression as the bacterium migrates from the lumen to approach the intestinal mucosa. Our findings also stress how a detailed understanding of the interaction of DSF ligands with VirF may contribute to the rational development of innovative antivirulence drugs to treat shigellosis.

## Introduction

*Shigella* is an enteropathogenic Gram-negative bacterium responsible for shigellosis, a disease that affects about 125 million people annually worldwide, with a fatal outcome in approximately 164,000 cases^1,2^. To infect the human intestinal mucosa, *Shigella* exploits a specific Type III Secretion System (T3SS) to promote bacterial invasion by injecting a series of factors into the host cell cytoplasm^3^. The genes encoding the protein component of this T3SS, the factors involved in its assembly, and the injectable effectors are clustered in a large pathogenicity island (PAI)-like region within the *Shigella* virulence plasmid (pINV)^4^. Expression of the T3SS genes is controlled by a regulatory cascade triggered by the AraC-XylS transcriptional regulator VirF, which sits at the top of the cascade^5,6^. The VirF protein promotes transcription of two genes critical for *Shigella* virulence, *icsA* and *virB.* The IcsA protein is involved in intracellular bacterial motility^7,8^ and the VirB protein, acting as the second positive regulator of the virulence system, is responsible for the expression of the underlying levels of the *Shigella* regulatory cascade^9,10^. Noteworthy, the *virF*, *virB* and *icsA* genes are pINV plasmid genes located outside the PAI-like region. VirF is regulated at the transcriptional level by several environmental stimuli, such as temperature, pH, and osmolarity, and at the post-transcriptional level by MiaA^10^. Very recently, we have shown that the activity of VirF is directly controlled also by fatty acids (FAs)^11^. In particular, FAs such as lauric, capric, myristoleic, palmitoleic and sapienic acid bind VirF and inactivate its transcription-promoting activity, probably by causing the protein structure to change from a free, functional (“open”) state to a FA-bound, non-functional (“closed”) form. A similar mechanism involving FAs has been proposed previously for other virulence regulators, as ToxT of *V. cholerae*, Rns of enterotoxigenic *E. coli* (ETEC), and HilD of *Salmonella enterica*^12–14^. Recently, cis-2-hexadecenoic acid was included among the ligands that bind and inhibit HilD, thus influencing virulence gene expression of *S. enterica*^14,15^. This compound belongs to a rare class of FAs, synthesised by Gram-negative bacteria, called “Diffusible signal factors” (DSFs). DSFs include FAs with different chain lengths and branching patterns with an unsaturated *cis* bond at position 2, except for 13-methyltetradecanoic acid (LeDSF). In addition, DSFs have been shown to serve as signalling molecules in bacterial quorum sensing (QS) systems^16,17^ and to regulate various functions in a wide range of bacteria, including plant and animal pathogens^17,18^. A prototype DSF molecule is cis-11-methyl-2-dodecenoic acid (XcDSF) produced by the phytopathogenic bacterium *Xanthomonas campestris*^17,19^. Other major, previously characterised members of the DSF family include cis-2-decenoic acid (PDSF); cis-2-dodecenoic acid (BDSF); cis, cis-11-methyldodeca-2, 5-dienoic acid (CDSF); cis-10-methyl-2-dodecenoic acid (IDSF); cis-2-tetradecenoic acid (XfDSF1); and cis-2-hexadecenoic acid (XfDSF2). Bacteria synthesizing DSFs are part of a constantly updated list and are characterized by the presence of the *rpfF* gene (or a homologue thereof), crucial for DSF synthesis^17,20^. To date, the list of DFS producers comprises *Xanthomonas spp*., *Xylella fastidiosa*, *Pseudomonas aeruginosa*, *Stenotrophomonas maltophilia*, *Lysobacter enzymogenes* and *brunescens*, *Leptospirillum ferrooxidans*, *Burkholderia cenocepacia* and *Cronobacter turicensis*^17^. Bacteria belonging to the genera *Frateuria*, *Luteobacter*, *Pseudoxhantomonas* and *Rhodanobacter* can be also included based on in silico prediction^17^. The gene locus responsible for both the production of DSFs and their recognition as quorum-sensing signal, has been characterized for the first time in *X. campestris* and includes four genes, *rpfC*, *rpfG*, *rpfB*, and *rpfF*^18,21,22^. RpfC and RpfG form a two-component system, while *RpfB* is a ligase for long-chain acyl-CoA molecules. RpfF is an enzyme of the crotonase superfamily (enoyl-CoA dehydratase and thioesterase activities) and acts on an acyl transporter protein (acyl-ACP), an intermediate of fatty acid synthesis, to synthesise XcDSF. When the bacterial population reaches a critical density, XcDSF interacts with the RpfC sensor inducing phosphorylation of the RpfG response regulator protein. Due to its hydrolytic activity the phosphorylated RpfG converts the second messenger bis-(3’-5’)-cyclic GMP (c-di-GMP) into GMP. A decrease in intracellular c-di-GMP leads to the deregulation of target genes^18^. In *Burkholderia cenocepacia* the biosynthesis of DSF molecules (BDSF) is entirely dependent on RpfFBc, a bifunctional enzyme with enoyl-CoA hydratase and thioesterase activity. This enzyme exhibits a significant sequence identity with the *X. campestris* RpfF protein. Despite the XcDSF and BDSF high similarity in the biosynthetic pathway and chemical structure, the mechanism by which *B. cenocepacia* sense BDSF differs from that characterized in *X. campestris* as it involves a cytoplasmic receptor, the RpfR protein. This enzyme exhibits cyclic di-GMP phosphodiesterase activity once bound to BDSF, thus causing cytoplasmic c-di-AMP to decrease^23^. Interestingly, this mechanism of recognition and regulation closely resembles the interaction between HilD and cis-2-hexadecenoic acid which causes suppression of *S. enterica* virulence. Considering the high structural similarity between XcDSF, BDSF, and lauric acid (Fig. S1), a previously characterized inhibitor of VirF activity^11^, we asked whether XcDSF and BDSF could inhibit *Shigella* virulence by preventing the transcription-promoting activity of the VirF protein. In the present study we show, by in silico, in vitro and in vivo experiments, that XcDSF and BDSF directly interact with VirF, leading to a significant decrease in *virB* transcription, thus hindering the expression of the entire virulence system of *Shigella* and causing a defective phenotype characterized by insufficient invasion of host cells and by delayed proliferation within them.

## Results

### VirF fails to derepress *virB* and *icsA* transcriptions in the presence of XcDSF and BDSF

**Figure 1:**
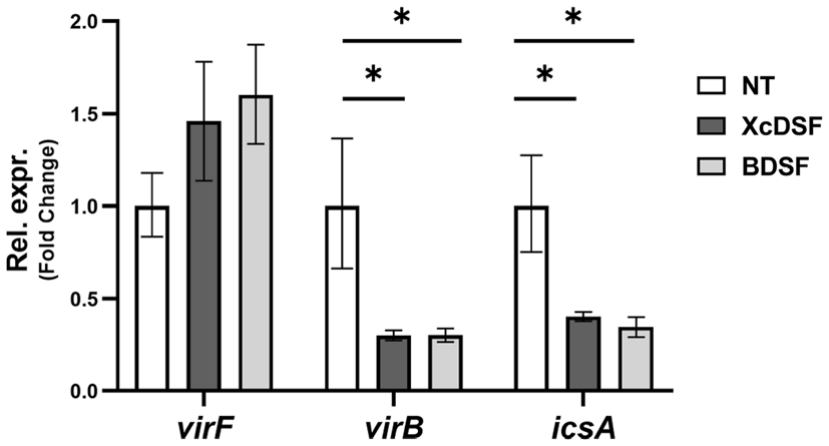
Comparative analysis of transcriptional profiles of *virF*, *virB* and *icsA* genes in *S. flexneri* after XcDSF or BDSF treatment. Comparative analysis of *virF*, *virB* and *icsA* gene transcriptional profiles in M90T *S. flexneri* strain grown in the presence or absence of 0.02% XcDSF and BDSF. Data are obtained by qRT-PCR and values are compared to the corresponding untreated sample set to 1. The results are an average of at least three independent experiments performed in triplicate. Error bars represent SD. Statistical significance was determined with a paired two-tailed Student’s *t* test using the measurements of the untreated and treated samples as a set of data. *p ≤ 0.01.

**Figure 2:**
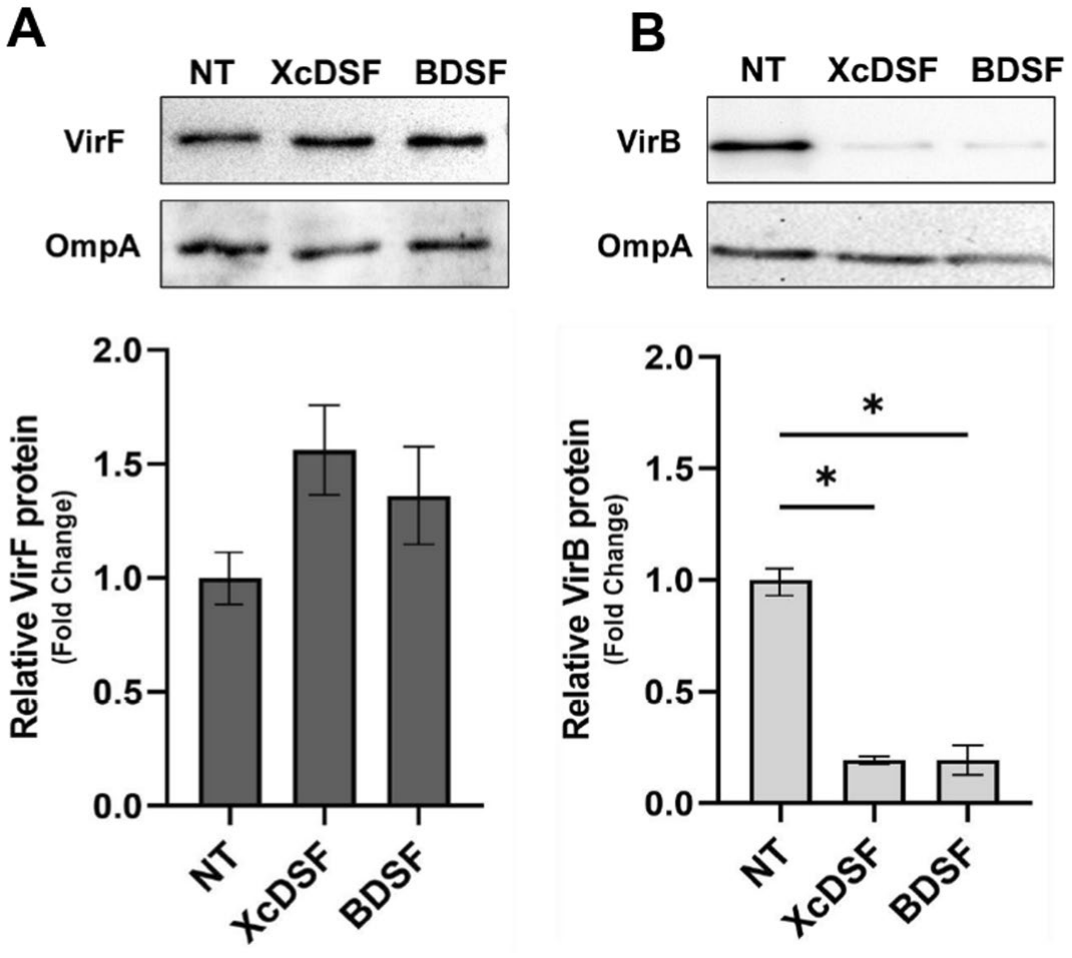
Comparative translational profiles of VirF and VirB protein in *S. flexneri* after XcDSF or BDSF treatment. Western blot analysis of VirF (**A**) and VirB (**B**) proteins in M90T *S. flexneri* strain grown in the absence or presence of 0.02% XcDSF and BDSF. Western blot images are representative of three independent experiments. All experiments’ densitometric analysis values were used to realize the bar graphs below the corresponding WB. The values were obtained by normalizing the VirF and VirB protein levels to those of the OmpA protein and are compared to the values of the untreated samples (NT) set to 1. Error bars represent SD. Statistical significance was determined with a paired two-tailed Student’s *t* test. *p ≤ 0.01.

To investigate the possible inhibitory effect of DSFs molecules on the *Shigella* virulence system, we initially determined the sub-inhibitory concentrations of XcDSF or BDSF on *Shigella* strain M90T growth in LB medium. The chain length of both DSFs is equivalent to that of lauric acid, which we have previously demonstrated to be an effective VirF inhibitor^11^. For this purpose, increasing concentrations of XcDSF or BDSF were added to M90T cultures to a final concentration of 0.2%, 0.02% or 0.002%, and growth was then monitored. The growth curves (Fig. S2A and B) clearly suggest that, for both DSFs, 0.02% is the highest concentration at which growth is not significantly influenced. Thus, by quantitative real-time PCR (qRT-PCR) following exposure of cultures to 0.02% XcDSF or BDSF we studied the transcription of *virF* and VirF-dependent *virB* gene. Moreover, we extended the analysis to the *icsA* gene to further confirm the possible inhibitory effect that XcDSF and BDSF may have on VirF activity to promote transcription. As compared to the untreated strain (NT), the relative level of *virF* transcript is slightly increased, whereas the level of *virB* and *icsA* transcripts is reduced by approximately 70% and 60–65% respectively (Fig. 1). To test whether the decreased transcription of the *virB* gene, crucial for the correct expression of the entire virulence system of *Shigella*, depends on negative modulation of VirF translation, we performed a Western blot assay with total protein extracts from *Shigella* M90T cultures expressing VirF-Flag-tagged (M90T VirF-FT) or VirB-Flag-tagged proteins (M90T VirB-FT) grown in the presence of XcDSF and BDSF. Comparative densitometric analysis of protein bands shows that the VirF (Fig. 2A) and VirB (Fig. 2B) protein levels are consistent with the transcriptional profiles, confirming that the amount of VirF protein is not affected by the presence of tested DSFs. Overall, these data suggest that XcDSF and BDSF impact *virB* transcription through the inhibition of the activity promoting transcription of VirF.

### Binding of XcDSF and BDSF to VirF inhibits its dimerization and subsequent binding to the *virB* promoter

**Figure 3:**
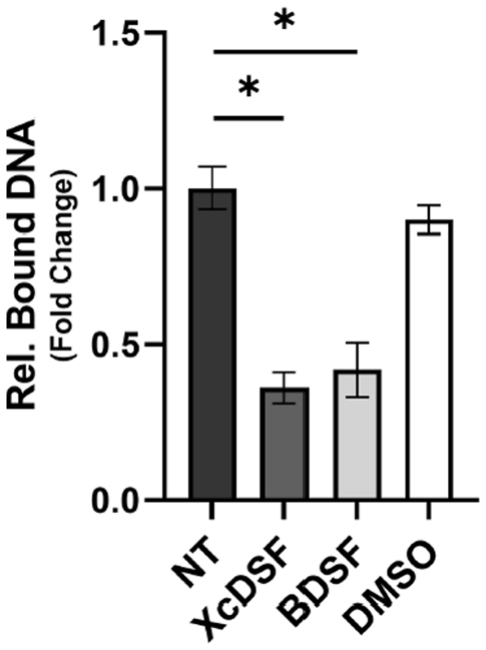
In vitro evaluation of VirF binding to the *virB* promoter in the presence or absence of XcDSF or BDSF. The bar graph shows the amount of fluorescence emitted by the P*virB* FITC-DNA retained in the wells by the VirF protein in the absence or presence of 0.02% XcDSF or BDSF. The experiment has been performed using saturating concentrations of P*virB* FITC-DNA fragment (75 ng) and the results are an average of at least three independent experiments, each performed in triplicate. Error bars represent SD. Statistical significance was determined with a paired two-tailed Student’s *t* test. *p ≤ 0.01.

**Figure 4:**
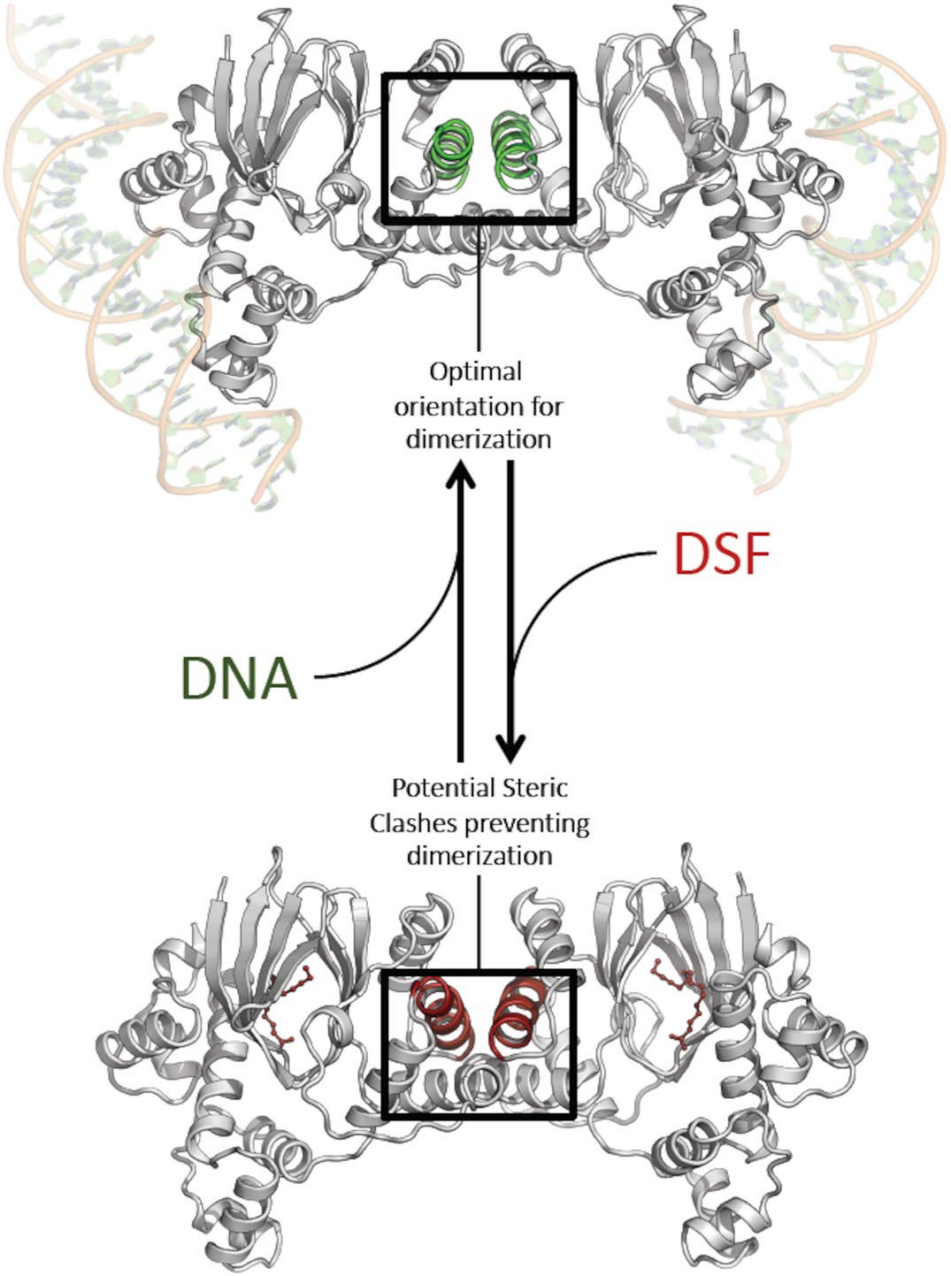
Proposed model of action of XcDSF or BDSF. The model hypothesized in the figure is based on the structural similarity between VirF and the other members of the AraC family. According to this hypothesis, the binding of XcDSF or BDSF to the jelly-roll domain is responsible for an allosteric reorientation of the helix α3, which in turn is part of the dimer interface of VirF. Such an allosteric transition prevents dimer formation and subsequent DNA binding.

Based on the data presented in the previous section, we hypothesise that XcDSF and BDSF, may act similarly to other fatty acids (FAs) we have recently studied^11^, i.e. they may interact directly with the VirF jelly roll module, a protein substructure composed of eight beta strands arranged in two four-stranded sheets, leading to a structural change and consequently to the inhibition of the VirF activity promoting transcription. We tested this hypothesis via a DNA–protein interaction plate assay (DPIPA)^11^ aimed at exploring the interaction of the purified VirF protein (MalE-VirF) with *PvirB*, a 194-bp FITC-tagged amplicon encompassing the proximal binding site of VirF to the *virB* promoter^24^. The results (Fig. 3) reveal that the relative ratio of binding of the MalE-VirF protein to *PvirB* DNA in the presence of XcDSF and BDSF is 0.38 and 0.37, respectively, as compared to untreated samples (set to1), indicating that the two DSFs clearly affect the interaction of VirF with the *virB* promoter. It has been demonstrated dimerization is an essential requirement for VirF (as well as for ToxT and Rns proteins) to bind DNA and activate the expression of target genes^25–28^. It is plausible that the binding of XcDSF or BDSF may influence the VirF dimerization. To explore this hypothesis, a molecular docking analysis was performed using the VirF model structure that we have previously predicted^11^. The structural alignment of the predicted XcDSF-bound VirF structure and the dimeric structure of the master regulator Rns from *E. coli* in apo form (PDB: 6XIU, 37% sequence identity)^13^ supports a mechanism of inhibition of VirF dimerization by the two DSFs tested (Fig. 4). In particular, the arrangement of the highly conserved α3 helix of VirF, which is homologous to the helix of Rns that forms the homodimer interface, is predicted at an angle that prevents dimerization when binding to the two DSFs occurs. Since the α3 helix of VirF is crucial for dimerization^25^, this observation suggests that XcDSF or BDSF binding could inhibit dimerization, and subsequent DNA-binding of VirF, by influencing the position of the α3 helix.

### VirF residues H17 and H212 are essential for the repressive action of XcDSF and BDSF

**Figure 5:**
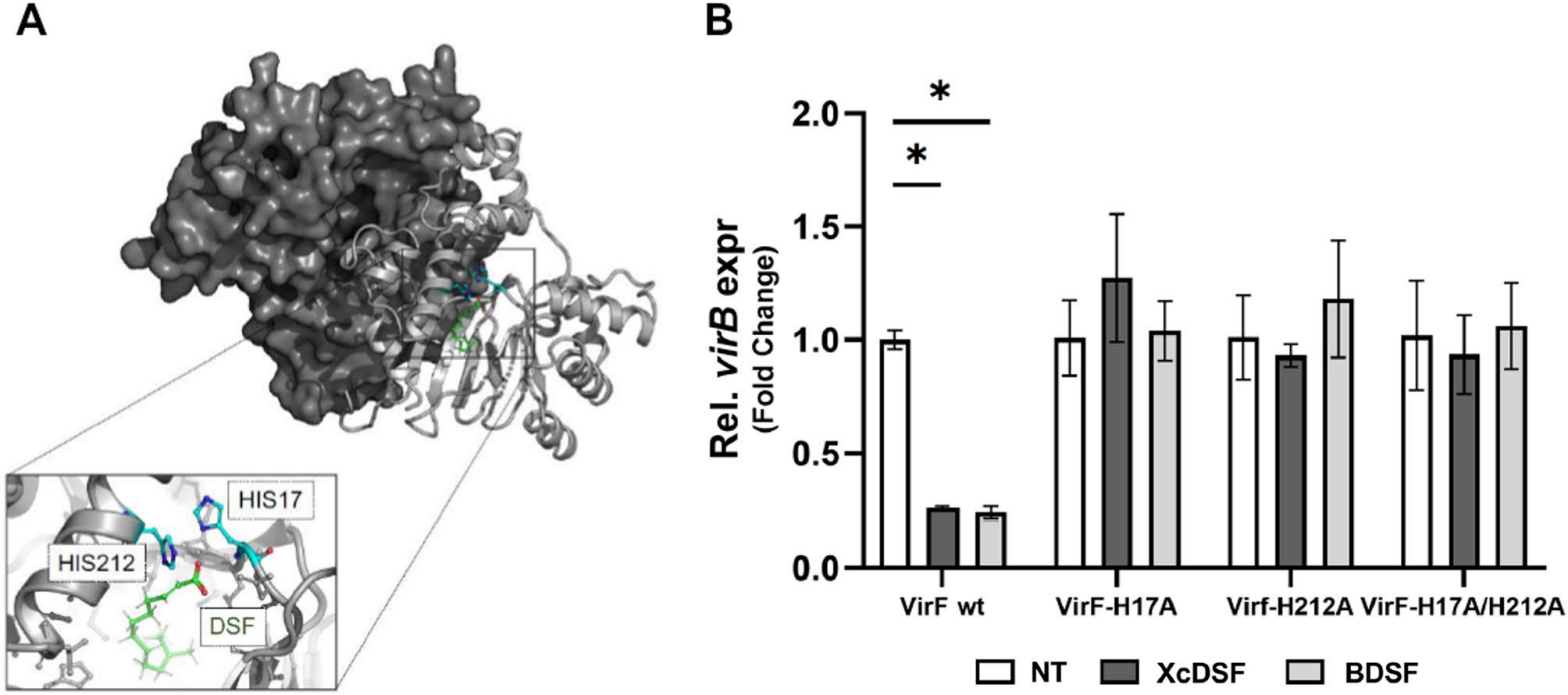
Role of H17 and H212 residues in the XcDSF and BDSF interaction with VirF protein. (**A**) The dimeric model of VirF is shown, with one monomer represented as a grey surface, and the other one as a white cartoon. XcDSF is represented as green sticks while the two histidine residues interacting with the ligand are cyan/blue sticks. The panel represents the cavity accommodating the ligand, and the histidine residues predicted to interact with the ligand are shown. (**B**) The graph shows the relative transcription of the *virB* gene in the presence of the VirF wild-type protein, the H17A, H212A, and H17A/H212A derivatives treated with 0.02% XcDSF (dark grey bars) or BDSF (light grey bars). The relative values were calculated using those from the corresponding untreated sample, set to 1. The results are an average of at least three independent experiments performed in triplicate. Error bars represent SD. Statistical significance was determined with a two-tailed Student’s *t* test using the set of measurements from the untreated and treated samples. *p ≤ 0.01.

To investigate whether the interaction of XcDSF and BDSF with the VirF protein also occurs in vivo, we evaluated *virB* transcription by qRT-PCR in a M90T Δ*virF* strain hosting the pVirFH17A, pVirFH212A, and pVirFH17A/212A plasmids (Table S3). These strains express mutated VirF proteins that are no longer sensitive to fatty acid inhibition, including lauric acid^11^. The molecular docking analysis clearly predicts that, similarly to the case of lauric acid, VirF residues H17 and H212 should be highly relevant to the XcDSF- or BDSF-VirF interaction (Fig. 5A). The outcome of qRT-PCR assays shows that the relative level of *virB* transcript in the strains carrying the mutated form of VirF and treated with XcDSF or BDSF is equivalent to that detected in untreated samples, while the level of *virB* transcript in the strain expressing the wild-type VirF protein is strongly decreased (to about 30%) (Fig. 5B). These observations confirm that both mutations impact *virB* regulation, abolishing the XcDSF-/BDSF-dependent repression, and together with the in vitro results they strongly suggest that the interaction of XcDSF and BDSF with VirF also occurs in vivo, causing the reduction of VirB expression and, therefore, of the entire virulence system.

### *Shigella* invasion of epithelial cells is reduced by XcDSF and BDSF treatment

**Figure 6:**
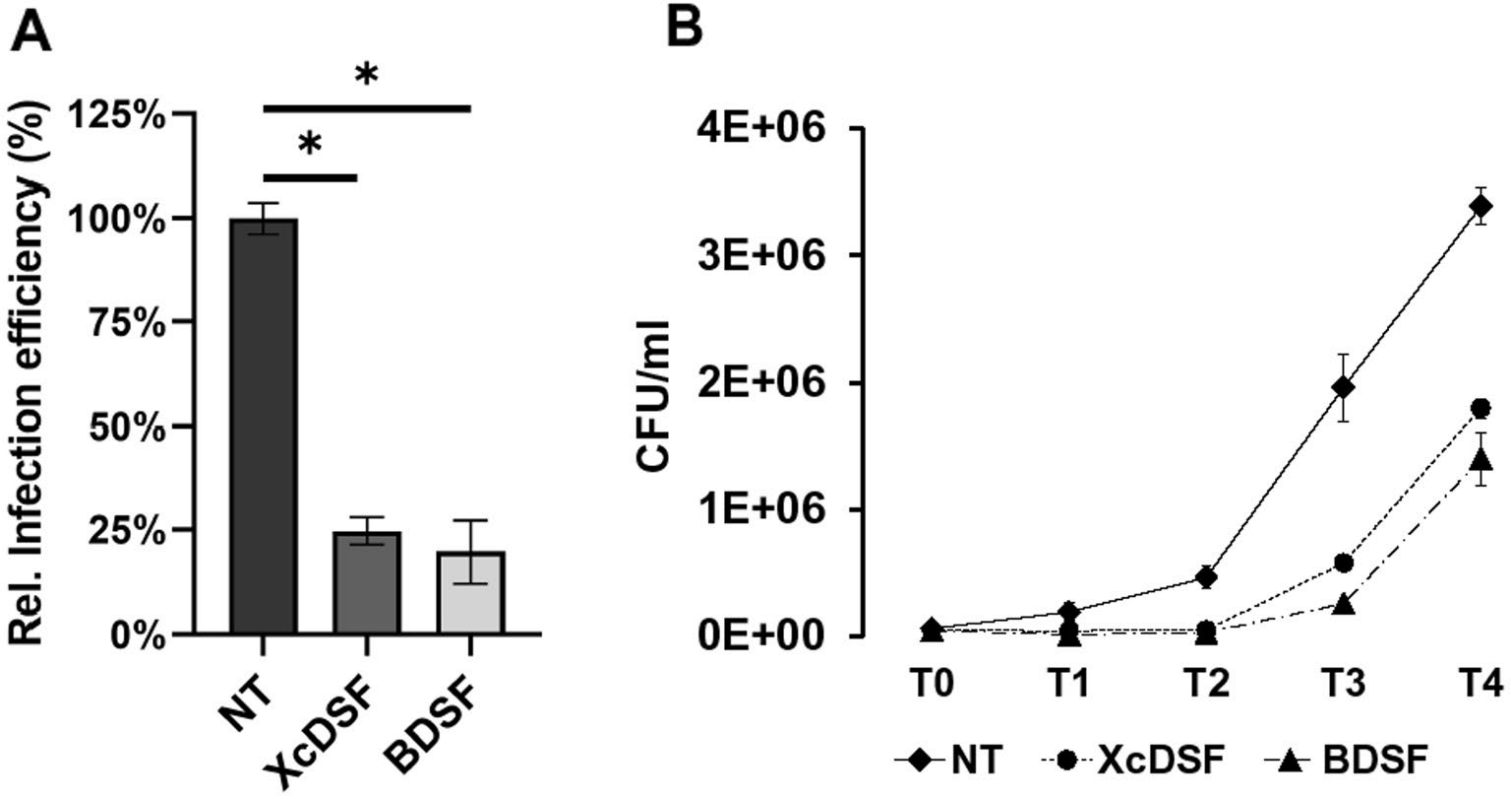
Evaluation of the *Shigella* entry and proliferation into the epithelial cell. Measurement of viable intracellular *S. flexneri* cells at T0 (**A**) or over 4 h of incubation p.i. (from T0 to T4) (**B**). The Caco-2 cell infection was carried out at MOI 100 with *S. flexneri* M90T grown in the absence (NT) or with 0.02% XcDSF or BDSF. The results are the average of at least three independent experiments. The statistical significance was determined with a two-tailed Student’s *t* test (**A**) or the two-way analysis of variance (ANOVA) (**B**) (p = 0.04). Error bars represent the SD.

Given the significant impact of XcDSF and BDSF on the virulence cascade of *Shigella*, we asked whether this was sufficient to affect its invasive phenotype and proliferative capability in epithelial cells. We therefore performed a gentamicin protection infection assay on a monolayer of Caco-2 epithelial cells, using the M90T *Shigella* strain grown in the presence of 0.02% XcDSF or BDSF. Allowing the infection to proceed for 4 h, viable intracellular bacteria were assessed at diverse timepoints (T0 to T4) by lysing the infected monolayers, and measuring bacterial viable count (CFU/ml). This allowed us to calculate the *Shigella* invasion (T0) and its intracellular proliferation (T1 to T4). The percentages of M90T bacteria invading Caco-2 cells after treatment with XcDSF or BDSF to T0, were 24% and 22% respectively (Fig. 6A). As for the intracellular proliferation, results clearly show that XcDSF- and BDSF-treated *Shigella* cells proliferate later in epithelial cells than untreated bacteria, with a delay of at least 1 hour (Fig. 6B). Altogether, these results indicate that treatment with XcDSF and BDSF has a significant impact on the entry of *Shigella* into epithelial cells, thus causing a delay in intracellular proliferation.

## Discussion

The microbial community of the human gut, also referred to as the human ‘microbiota’, consists of nearly 3000 microbial species, mainly bacteria (90%)^29^. Their density and diversity progressively increase throughout the gastrointestinal tract. The upper parts of the intestine, the stomach, and the proximal duodenum, are normally colonised only by relatively simple microbial communities that can withstand acidic conditions and the presence of bile and pancreatic enzymes. Higher microbial density and diversity are found in the colon, where transit time is slower and there is greater availability of nutrients^30^. The gut microbiota contributes to many important functions of the human body, including digestion, gut endocrine function, neurological signalling, and immune system formation and development. It also contributes to the integrity of the intestinal mucosa, provides vitamins and nutrients, and counteracts colonisation by pathogens^31–33^. The bacterial components of the human microbiota use quorum sensing (QS) to communicate with each other. This mechanism, based on synthesis, release, recognition, and response to signalling molecules, called autoinducers, enables the coordination of bacterial activities in the gut, not only among different microbial species but also concerning their interaction with the host^34^. In the human gut, autoinducers produced by commensal and pathogenic bacteria have been identified and linked to gut health and disease^35,36^. Autoinducers synthesized by commensal bacteria have been shown to help maintain intestinal barrier integrity, modulate inflammatory processes, and promote “colonization resistance”^37^, i.e. the processes depending on resident microorganisms acting against enteric pathogens based on competition for nutrients, metabolic exclusion, O_2_ consumption, bacteriocin or antimicrobial peptide production, and QS interference^38–40^. Bacterial pathogens use their autoinducers to coordinate the expression of virulence factors and influence the host response^41^. In addition, bacterial pathogens can eavesdrop on host- and commensal-derived signalling molecules to spatially orient themselves and optimise virulence expression at the site of infection^38,42^. In this work, we show that in *Shigella* sub-millimolar concentrations of XcDSF and BDSF, two quorum-sensing signals belonging to the DSF family, can elicit a significant decrease of *virB* transcription (Fig. 1). In particular, our observations (Figs. 2, 3, 5) indicate that this depends on XcDSF- or BDSF-mediated inhibition of the VirF activation of the *virB* gene promoter resulting from direct interaction of the two DSF molecules with the VirF protein itself. Furthermore, based on molecular modelling analyses, we propose a mechanism where binding of XcDSF or BDSF to the VirF protein causes switching of the VirF α3 helix into a position no longer allowing dimerization (Fig. 4). Lastly, results from *Shigella* infection assays of Caco-2 epithelial cells indicate that XcDSF- or BDSF-mediated suppression of VirF activity significantly inhibits *Shigella* invasion of host cells and intracellular proliferation (Fig. 6). Altogether, these observations demonstrate that XcDSF and BDSF, the two most representative QS molecules of the DSF family, exert a significant anti-virulence effect on *Shigella*. Our results are in line with those obtained previously in *S. enterica*, where another member of the DSF family, cis-2-hexadecenoic acid (c2-HDA or XfDSF2), binds the HilD protein, the main transcriptional regulator of *Salmonella*, preventing its dimerization and activity to promote transcription, and therefore *Salmonella* patogenicity^15^. VirF and HilD belong to the same subclass of AraC-XylS family protein^43^ and share the inhibitory mechanism mediated by fatty acid binding^11,44^. Given that, and based on the results of this work, we can speculate that other compounds of the DSF family may be candidates as inhibitors of VirF, HilD and even for other proteins of the same AraC-XylS subfamily, including ToxT in *Vibrio cholerae* and Rns in enterohaemorrhagic *E. coli* (ETEC). Nevertheless, considering that the inhibition seems to be influenced by the aliphatic chain length of the DSF compounds (Bosire et al.^14^), and based on the differences in the inhibitory capacity of the fatty acids of HilD and VirF (e.g. palmitoleic acid)^11,14^, the inhibition of DSF molecules of cited AraC-XylS proteins may not be completely comparable. Although the interactions described so far between the master regulators of the virulence mechanisms of typically human enteropathogens suggests that the DSF molecules are present in the human gut, this is still debated. Nevertheless, some additional evidence supports this presence. In particular, the identification of the DSF producer *Stenotrophomonas maltophilia* as part of the central crypt-specific microbiota of the mouse gut^45,46^ and the evaluation of cis-2-hexadecenoic acid (c2-HDA) as one of the most abundant molecules in the fatty acid fraction of mouse colonic contents^15^, show that DSFs are present in the mouse gut and suggest that they may be present also in the gut of other mammals, including humans. Moreover, in silico predictions have shown the existence of DSF genetic circuits in many human gut microorganisms^47,48^. In conclusion, given the probable presence of DSFs in the human gut, we can speculate that DSFs may contribute to “colonisation resistance” by inhibiting the virulence of pathogens such as *V. cholerae, S. enterica,* and *S. flexneri*^15^. Alternatively, DSFs could participate in the fine tuning of virulence expression in enteropathogens by preventing the full expression of virulence systems in the intestinal lumen, where DSFs might be present at higher levels, and allowing their expression as the bacterium approaches the intestinal mucosa, where luminal compounds are present in lower concentrations^12^. In this regard, it is interesting to note that when *Shigella* approaches the intestinal mucosa, in addition to experiencing a decrease in the concentration of DSF molecules, leading to a derepression of the virulence, it also perceives an increase in oxygenation depending on the presence of the capillary network in the mucosa. This leads to full activation of the T3SS, thus increasing virulence^49^. We can therefore hypothesise that the dilution of DSF molecules and the increase in oxygen cooperate to optimise the expression of *Shigella* virulence at the correct site of action. Overall, the characterization of the interaction between VirF and DSF molecules may contribute to the rational development of innovative compounds exhibiting anti-virulence activity by specifically targeting bacterial virulence without interfering with cell growth. This could provide therapeutic strategies other than the use of antibiotics in the treatment of shigellosis, thus curbing the alarming increase in antibiotic-resistant *Shigella* strains isolated in recent years^50^.

## Methods

### Bacterial strains, general growth conditions and DNA methods

The bacterial strains used in this study are listed in Table S1. Bacterial cells were grown aerobically in Luria–Bertani (LB) medium (Sigma-Aldrich) at 37 °C and, when required, antibiotics were added at the following final concentrations: ampicillin 100 μg/mL; kanamycin 30 μg/ml; streptomycin 10 μg/ml. To evaluate the effect of XcDSF and BDSF (Sigma-Aldrich) on bacterial growth, a 5% solution of these compounds was obtained by heating the tube to 37 °C and shaking it in the ultrasonic bath. This solution was then diluted to the indicated final concentrations in LB medium, taking care to keep the concentration of DMSO (4%) in each sample constant. To perform the other in vivo and in vitro experiments, we used a 2% solution of XcDSF or BDSF in DMSO which was then diluted in LB, or in a dedicated buffer, to a final concentration of 0.02%. The oligonucleotide sequences, designed based on the M90T genome, are reported in Table S2. PCR reactions were routinely performed using the DreamTaq DNA polymerase (Thermo Fisher Scientific) or when required a higher fidelity of PCR product, the Ex Taq DNA-polymerase (Takara). Plasmids used in this work are listed in Table S3.

### RNA isolation and quantitative real-time PCR

Bacterial RNA purification was performed as previously described^51^ and cDNA synthesis was obtained with the High-Capacity cDNA Reverse Transcription Kit (Applied Biosystems). Briefly, 1.5 μg of total RNA from bacteria was treated with DNAse I and then retro-transcribed in a 20 μl reaction mix following the manufacturer’s instructions. Quantitative real-time PCR (qRT-PCR) analysis was performed on a StepOnePlus™ Real-Time PCR System (Applied Biosystem). The reaction volume was 20 μl containing Power SYBR Green PCR Master Mix (Applied Biosystem), 2 μl of cDNA sample, and specific oligos for the *virF*, *virB*, *icsA* and *nusA* genes (300 nM each), the last used as an endogenous control. The cycling conditions were as follows: 1 cycle 95 °C for 2 min, 40 cycles 95 °C for 10 s followed by 60 °C for 30 s. The quantitative analysis of the transcripts was based on the 2-ΔΔCt method^52^, and the results are indicated as “relative expression” to the reference sample. All the oligo pairs used for qRT-PCR, designed using the Primer Express software v2.0, are reported in Table S2 and were experimentally validated.

### SDS-PAGE and immunoblot analysis

Bacteria pellets were resuspended in PBS with 1X Final Sample Buffer (FSB) and, after boiling at 100 °C, loaded on 12.5% SDS-PAGE. A protein molecular weight marker (Page ruler; Thermo Fisher) was included in each electrophoresis run. Proteins were transferred to nitrocellulose membranes (Hybond-P, Millipore), and the immunoblotting was carried out with Monoclonal ANTI-FLAG® M2 antibody (Sigma-Aldrich) and rabbit polyclonal anti-OmpA^53,54^ as primary antibodies, and HRP conjugated goat anti-mouse and anti-rabbit IgG antibody as the secondary antibodies (Sigma-Aldrich). Signals were produced with ECL Star (Euroclone) and detected with ChemiDoc™ Gel Imaging System (Bio-Rad Laboratories). The densitometric analysis was performed by ImageJ software^55^.

### Purification of MalE-VirF

Overnight cultures of *E. coli* XL1BLUE pMALcF1 were diluted 100-fold in 1 L LB medium containing 0.2% glucose and incubated at 37 °C. Glucose was necessary to repress maltose chromosomal genes and prevent the expression of amylase, thus amylose degradation on affinity resin. When the culture density reached an OD600 of 0.5, 0.3 mM IPTG was added to induce the *malE*-*virF* gene transcription and incubation was continued for an additional 4 h at 37 °C. Cells harvested by centrifugation were resuspended in 10 ml of buffer A (20 mM Tris–HCl, pH 7.5, 1 mM EDTA, and 200 mM NaCl). Resuspended cells were disrupted by sonication on ice and centrifuged at 12,000 rpm at 4 °C for 20 min to remove cell debris. The supernatant was diluted fivefold with buffer A and loaded onto a 5-ml column of amylose resin (New England Biolabs) preequilibrated in buffer A at a flow rate of 1 ml/min. The column was washed with 40 ml of buffer A and the fusion protein was eluted with 20 ml of buffer A containing 10 mM maltose. Fractions containing MalE-VirF, as judged by SDS-PAGE analysis, were pooled, washed (to remove maltose), and concentrated with filter devices with 50KDa MWCO (Millipore). Protein concentration was calculated using a theoretical extinction coefficient at 280 nm of 89,730 M^−1^ cm^−1^ (calculated with the Expasy ProtParam tool).

### DNA–protein interaction plate assay (DPIPA)

The DPIPA assay^11^ uses the dextrin-coated flat-bottomed surface of polystyrene microplate wells (Nunc MaxisorpTM, Thermo Fisher Scientific) as a solid matrix for affinity adsorption of the MalE-VirF protein. This was then used to measure the amount of the P*virB* DNA fragment comprising the proximal VirF binding site on the *virB* promoter sequence^24^ and obtained by PCR amplification using the pvirB_F FITC/pvirB_R FITC oligo pair and pBN1 plasmid as the template. Briefly, 96 plate wells were treated with 150 μl of 0.5% wt/vol dextrin from maize starch (Sigma-Aldrich) dissolved in 100 mM sodium phosphate buffer pH 7 (PBS), overnight at 4 °C in a humid chamber. The wells were then blocked for 1 h with 150 μl of 1.5% bovine serum albumin (BSA) in PBS at 37 °C. After washing with PBS, 50 μl of Binding buffer solution (100 mM Tris–HCl pH 7.5, 1 mM DTT, 50 mM NaCl, 0.1% NP-40) containing 1 μM of MalE-VirF protein, 75 ng of FITC-labelled PCR DNA fragments and, when required, 0.02% XcDSF or BDSF, were added to the wells and incubated at room temperature for 30 min with gentle agitation. Finally, the supernatant was removed, and the fluorescence of bound DNA was detected with the CLARIOstar microplate reader (BMG LABECH, Offenburg, Germany).

### Molecular modeling

Modeling and docking procedures were performed as already described^11^. Briefly, the dimeric model of VirF was obtained using AlphaFold2 (version 2.0.1)^56^ with template-based homologous transcriptional regulator Rns (https://www.rcsb.org/; PDB accession no. 6XIU; percentage of identity, 41%)^13^. The docking of DSF into the active site was performed using the Molegro Virtual Docker (MVD) software v7.0 (Molexus). DNA-binding of VirF was predicted using as template the structure of Mar-A (PDB: 1XS9^54^). Structure modeling and analysis were carried out using PyMod 3.0^57^. The conformational transitions of VirF between the open and closed conformations were obtained using the Elastic Network Model, as implemented in PyANM^58^, with default values.

### Cell cultures and infections

Infection experiments were performed by using Caco-2 cell lines as previously described^59^. Human Caco-2 epithelial cells (American Type Culture Collection, Manassas, VA) were grown at 37 °C in a humidified 5% CO_2_ atmosphere in DF10, which comprises 10% heat-inactivated Fetal Bovine Serum FBS (FBS), 2 mM l-glutamine and 10% Penicillin–Streptomycin solution (PS) (Sigma-Aldrich) in Dulbecco modified essential medium (DMEM) (GIBCO). For bacterial infection, cells were seeded in 6-well tissue culture plates (Falcon), at a density of 4 × 10^5^ cells/well, in DF10. After 24 h, cells were serum-starved overnight in a modified DF10 medium with 0.5% FBS (DF0.5). DF0.5 was replaced with fresh DMEM containing only l-glutamine 2 hours before bacterial infection. The cell line was infected at a MOI of 100. After the addition of untreated and 0.02% XcDSF- or BDSF-treated bacteria, plates were centrifuged for 15 min at 750 ×*g* and incubated for 45 min at 37 °C under a 5% CO_2_ atmosphere to allow bacterial entry. Afterwards, extracellular bacteria were removed by complete washing with phosphate-buffered saline (PBS). This point was considered as time zero (T0). Fresh DMEM medium containing gentamicin (100 μg/ml) was added to each plate to kill extracellular bacteria, and the infected cells were incubated at 37 °C for up to 4 h. To calculate the number of intracellular bacteria for all infection times, cells were lysed, and bacteria were recovered, washed with 0.9% NaCl and serially diluted on LB agar. The percentage of bacteria that penetrated Caco-2 epithelial cells was calculated by comparing the CFU/ml at time T0 with those obtained by plating the bacterial load used to infect the epithelial cells (i.e. the bacterial pre-infection control). Bacterial survival was analysed by calculating the CFU/ml obtained over 4 h p.i. (T0–T4).

### Statistical analysis

Statistical significance was determined with the two-tailed Student’s *t* test in RT-PCR experiments, densitometric analysis of Western Blot and DPIPA. The two-way analysis of variance (ANOVA) was used to calculate the statistical significance of the results regarding the invasion ability of *Shigella* in Caco-2 cells.

### Supplementary Information

Supplementary Information.

## Data Availability

The datasets analysed during this study are available from the corresponding author on reasonable request.

## References

[1] Kotloff KL, Riddle MS, Platts-Mills JA, Pavlinac P, Zaidi AKM (2018). Shigellosis. Lancet.

[2] The HC, Thanh DP, Holt KE, Thomson NR, Baker S (2016). The genomic signatures of Shigella evolution, adaptation and geographical spread. Nat. Rev. Microbiol..

[3] Schnupf P, Sansonetti PJ (2019). *Shigella* pathogenesis: New insights through advanced methodologies. Microbiol. Spectr..

[4] Parsot C (2005). Shigella spp. and enteroinvasive *Escherichia*
*coli* pathogenicity factors. FEMS Microbiol. Lett..

[5] Falconi M, Colonna B, Prosseda G, Micheli G, Gualerzi CO (1998). Thermoregulation of Shigella and *Escherichia*
*coli* EIEC pathogenicity. A temperature-dependent structural transition of DNA modulates accessibility of virF promoter to transcriptional repressor H-NS. EMBO J..

[6] Prosseda G (2004). The virF promoter in Shigella: More than just a curved DNA stretch. Mol. Microbiol..

[7] Bernardini ML, Mounier J, D’Hauteville H, Coquis-Rondon M, Sansonetti PJ (1989). Identification of icsA, a plasmid locus of *Shigella*
*flexneri* that governs bacterial intra- and intercellular spread through interaction with F-actin. Proc. Natl. Acad. Sci. U. S. A..

[8] Tran CN (2011). A multifactor regulatory circuit involving H-NS, VirF and an antisense RNA modulates transcription of the virulence gene icsA of *Shigella*
*flexneri*. Nucleic Acids Res..

[9] Beloin C, McKenna S, Dorman CJ (2002). Molecular dissection of VirB, a key regulator of the virulence cascade of *Shigella*
*flexneri*. J. Biol. Chem..

[10] Di Martino ML, Falconi M, Micheli G, Colonna B, Prosseda G (2016). The multifaceted activity of the VirF regulatory protein in the Shigella Lifestyle. Front. Mol. Biosci..

[11] Trirocco R (2023). Fatty acids abolish Shigella virulence by inhibiting its master regulator, VirF. Microbiol. Spectr..

[12] Mitchell MK, Ellermann M (2022). Long chain fatty acids and virulence repression in intestinal bacterial pathogens. Front. Cell. Infect. Microbiol..

[13] Midgett CR, Talbot KM, Day JL, Munson GP, Kull FJ (2021). Structure of the master regulator Rns reveals an inhibitor of enterotoxigenic *Escherichia*
*coli* virulence regulons. Sci. Rep..

[14] Bosire EM (2020). Diffusible signal factors act through AraC-type transcriptional regulators as chemical cues to repress virulence of enteric pathogens. Infect. Immun..

[15] Chowdhury R, Bitar PDP, Keresztes I, Condo AM, Altier C (2021). A diffusible signal factor of the intestine dictates Salmonella invasion through its direct control of the virulence activator HilD. PLoS Pathog..

[16] Wang LH (2004). A bacterial cell–cell communication signal with cross-kingdom structural analogues. Mol. Microbiol..

[17] He YW (2023). DSF-family quorum sensing signal-mediated intraspecies, interspecies, and inter-kingdom communication. Trends Microbiol..

[18] Dow J, John Maxwell Dow C (2017). Diffusible signal factor-dependent quorum sensing in pathogenic bacteria and its exploitation for disease control. J. Appl. Microbiol..

[19] Yu YH, Hu Z, Dong HJ, Ma JC, Wang HH (2016). *Xanthomonas*
*campestris* FabH is required for branched-chain fatty acid and DSF-family quorum sensing signal biosynthesis. Sci. Rep..

[20] Kumar P, Lee JH, Beyenal H, Lee J (2020). Fatty acids as antibiofilm and antivirulence agents. Trends Microbiol..

[21] Barber CE (1997). A novel regulatory system required for pathogenicity of *Xanthomonas*
*campestris* is mediated by a small diffusible signal molecule. Mol. Microbiol..

[22] Zhou L, Zhang LH, Cámara M, He YW (2017). The DSF family of quorum sensing signals: Diversity, biosynthesis, and turnover. Trends Microbiol..

[23] Deng Y (2012). Cis-2-dodecenoic acid receptor RpfR links quorum-sensing signal perception with regulation of virulence through cyclic dimeric guanosine monophosphate turnover. Proc. Natl. Acad. Sci. U. S. A..

[24] Ragazzone NJ, Dow GT, Garcia GA (2022). Elucidation of key interactions between VirF and the virB promoter in *Shigella*
*flexneri* using *E*. *coli* MarA- and GadX-based homology models and in vitro analysis of the DNA-binding domains of VirF and MarA. J. Bacteriol..

[25] Dow GT, Young AM, Garcia GA (2023). Elucidation of the DNA-binding activity of VirF from *Shigella*
*flexneri* for the icsA and rnaG promoters and characterization of the N-terminal domain to identify residues crucial for dimerization. J. Bacteriol..

[26] Prouty G (2005). Characterization of functional domains of the Vibrio cholerae virulence regulator ToxT. Mol. Microbiol..

[27] Childers BM (2011). N-terminal residues of the vibrio cholerae virulence regulatory protein ToxT involved in dimerization and modulation by fatty acids. J. Biol. Chem..

[28] Mahon V, Fagan RP, Smith SGJ (2012). Snap denaturation reveals dimerization by AraC-like protein Rns. Biochimie.

[29] Stamboulian M, Doak TG, Ye Y (2020). A tree of human gut bacterial species and its applications to metagenomics and metaproteomics data analysis. bioRxiv.

[30] Dieterich W, Schink M, Zopf Y (2018). Microbiota in the Gastrointestinal Tract. Med. Sci..

[31] Valdes AM, Walter J, Segal E, Spector TD (2018). Role of the gut microbiota in nutrition and health. BMJ.

[32] Ma N (2018). Nutrients mediate intestinal bacteria-mucosal immune crosstalk. Front. Immunol..

[33] Altveş S, Yildiz HK, Vural HC (2020). Interaction of the microbiota with the human body in health and diseases. Biosci. Microbiota Food Heal..

[34] Zhang Y, Ma N, Tan P, Ma X (2022). Quorum sensing mediates gut bacterial communication and host-microbiota interaction. Crit. Rev. Food Sci. Nutr..

[35] Coquant G (2021). Gossip in the gut: Quorum sensing, a new player in the host-microbiota interactions. World J. Gastroenterol..

[36] Uhlig F, Hyland NP (2022). Making sense of quorum sensing at the intestinal mucosal interface. Cells.

[37] Yang Y (2022). Within-host evolution of a gut pathobiont facilitates liver translocation. Nature.

[38] Bäumler AJ, Sperandio V (2016). Interactions between the microbiota and pathogenic bacteria in the gut. Nature.

[39] Rolhion N, Chassaing B (2016). When pathogenic bacteria meet the intestinal microbiota. Philos. Trans. R. Soc. B Biol. Sci..

[40] Thursby E, Juge N (2017). Introduction to the human gut microbiota. Biochem. J..

[41] Dicks LMT (2022). How does quorum sensing of intestinal bacteria affect our health and mental status?. Microorganisms.

[42] Markus V (2023). Conversations in the gut: The role of quorum sensing in normobiosis. Int. J. Mol. Sci..

[43] Cortés-Avalos D (2021). An update of the unceasingly growing and diverse AraC/XylS family of transcriptional activators. FEMS Microbiol. Rev..

[44] Golubeva YA, Ellermeier JR, CottChubiz JE, Slauch JM (2016). Intestinal long-chain fatty acids act as a direct signal to modulate expression of the Salmonella pathogenicity island 1 type III secretion system. MBio.

[45] Pédron T (2012). A crypt-specific core microbiota resides in the mouse colon. MBio.

[46] Saffarian A (2019). Crypt- and mucosa-associated core microbiotas in humans and their alteration in colon cancer patients. MBio.

[47] Wu S (2022). Machine learning aided construction of the quorum sensing communication network for human gut microbiota. Nat. Commun..

[48] Falà AK, Álvarez-Ordóñez A, Filloux A, Gahan CGM, Cotter PD (2022). Quorum sensing in human gut and food microbiomes: Significance and potential for therapeutic targeting. Front. Microbiol..

[49] Marteyn B (2010). Modulation of Shigella virulence in response to available oxygen in vivo. Nature.

[50] Puzari M, Sharma M, Chetia P (2018). Emergence of antibiotic resistant Shigella species: A matter of concern. J. Infect. Public Health.

[51] Prosseda G (2006). Plasticity of the Pjunc promoter of ISEc11, a new insertion sequence of the IS1111 family. J. Bacteriol..

[52] Livak KJ, Schmittgen TD (2001). Analysis of relative gene expression data using real-time quantitative PCR and the 2−ΔΔCT method. Methods.

[53] Pasqua M (2021). Modulation of omv production by the lysis module of the dlp12 defective prophage of escherichia coli k12. Microorganisms.

[54] Scribano D (2014). Polar localization of PhoN2, a periplasmic virulence-associated factor of *Shigella*
*flexneri*, is required for proper IcsA exposition at the old bacterial pole. PLoS One.

[55] Schindelin J, Rueden CT, Hiner MC, Eliceiri KW (2015). The ImageJ ecosystem: An open platform for biomedical image analysis. Mol. Reprod. Dev..

[56] Jumper J (2021). Highly accurate protein structure prediction with AlphaFold. Nature.

[57] Janson G, Paiardini A (2021). PyMod 3: A complete suite for structural bioinformatics in PyMOL. Bioinformatics.

[58] Atilgan AR (2001). Anisotropy of fluctuation dynamics of proteins with an elastic network model. Biophys. J..

[59] Leuzzi A (2017). Role of the SRRz/Rz1 lambdoid lysis cassette in the pathoadaptive evolution of Shigella. Int. J. Med. Microbiol..

