# Supplementary material for: Diffusible signal factors (DSFs) bind and repress VirF, the leading virulence activator of Shigella flexneri
Source: Sci Rep. 2023 Aug 14;13:13170. doi: 10.1038/s41598-023-40023-w (PMC10425336; doi:10.1038/s41598-023-40023-w)

Diffusible Signal Factors (DSFs) bind and repress VirF, the leading virulence activator of *Shigella flexneri*

Rita Trirocco, Martina Pasqua, Angela Tramonti, Bianca Colonna, Alessandro Paiardini, Gianni Prosseda *.

Supplemental material

Tables

Table S1
Strains used in this work.

Strain	Characteristic	Source/References
XL1BLUE	*recA1 endA1 gyrA96 thi-1 hsdR17 supE44 relA1 lac [F proAB lacIqZΔM15 Tn10 (Tetr)]*	Agilent Technologies
M90T	*S.flexeneri* 5a; wild type harbouring 220-kb invasion plasmid pWR 100	1
M90T F-FT	M90T with VirF-tagged (3xFLAG) protein	2
M90T B-FT	M90T with VirB-tagged (3xFLAG) protein	2
M90T Fd	M90T with *virF* gene deletion (Δ *virF*)	3

Table S2

Oligos used in this work.

Name	5′-3′ sequence
qVirF F	AAAGGTGTTCAATGACGGTTAGC
qVirF R	CAATTTGCCCTTCATCGATAGTC
qVirB F	GGAAGGCCAAAAGAAAGAGTTTACA
qVirB R	GAGGAATCTTGGCTTTGATAAAGG
qicsA F	TGATGGACTTTCTCCCTTGGG
qicsA R	TACCACGCATCCATTCCATCT
qNusA F	CGTAGGCACATTCGAACAACG
qNusA R	CCTGGTACAGCAAGTTGCG
pvirB_F FITC	5′FITC - GGAGCTCTCACATCAGAGCTCCAC
PvirB R FITC	5′FITC - CCATATCACACCCTGTTTATTCATATTG

Table S 3
Plasmid used in this work.

Plasmids	Characteristic	Source/References
pMALcF1	pMALc2x derivatives containing the *virF* gene	This study
pBN1	pBR322-derived vector carrying the *virB* gene	4
pMYSH6504	pBR322 derivative carrying the *Shigella virF* gene	5
pVirFH17A	pMYSH6504 derivative carrying the*virF* mutated gene (cat -> gcg; +110 +112)	3
pVirFH212A	pMYSH6504 derivative carrying the*virF* mutated gene (cat -> gcg; +695 +697)	3
pVirFH17A/H212A	pMYSH6504 derivative carrying the *virF* mutated gene (cat -> gcg +110 +112 and cat -> gcg +695 +697)	3

References

1. Sansonetti PJ, Kopecko DJ, Formal SB. Involvement of a plasmid in the invasive ability of Shigella flexneri. Infect Immun. 1982 Mar;35(3):852-60. doi: 10.1128/iai.35.3.852-860.1982. PMID: 6279518; PMCID: PMC351125.
2. Di Martino ML, Romilly C, Wagner EG, Colonna B, Prosseda G. One Gene and Two Proteins: a Leaderless mRNA Supports the Translation of a Shorter Form of the Shigella VirF Regulator. mBio. 2016 Nov 8;7(6):e01860-16. doi: 10.1128/mBio.01860-16. PMID: 27834204; PMCID: PMC5101355.
3. Trirocco R, Pasqua M, Tramonti A, Grossi M, Colonna B, Paiardini A, Prosseda G. Fatty Acids Abolish Shigella Virulence by Inhibiting Its Master Regulator, VirF. Microbiol Spectr. 2023 Jun 15;11(3):e0077823. doi: 10.1128/spectrum.00778-23. Epub 2023 May 4. PMID: 37140433; PMCID: PMC10269687.
4. Uzzau S, Figueroa-Bossi N, Rubino S, Bossi L. Epitope tagging of chromosomal genes in Salmonella. Proc Natl Acad Sci U S A. 2001 Dec 18;98(26):15264-9. doi: 10.1073/pnas.261348198. Epub 2001 Dec 11. PMID: 11742086; PMCID: PMC65018.
5. Sakai T, Sasakawa C, Makino S, Yoshikawa M. DNA sequence and product analysis of the virF locus responsible for congo red binding and cell invasion in Shigella flexneri 2a. Infect Immun. 1986 Nov;54(2):395-402. doi: 10.1128/iai.54.2.395-402.1986. PMID: 3021627; PMCID: PMC260174.

Figure

Figure S1.

Chemical structure of lauric acid, XcDSF and BDSF.

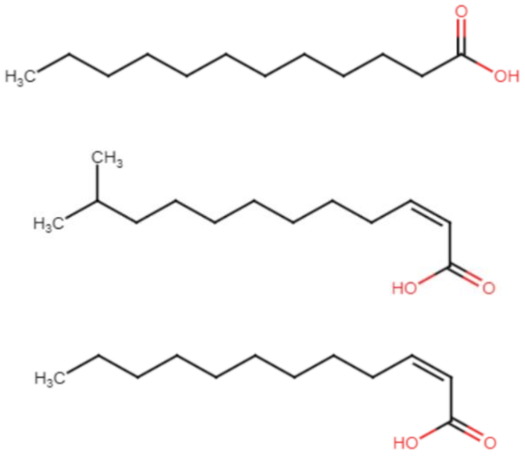

Lauric

XcDSF

BDSF

Lauric acid, XcDSF and BDSF have the same carbon chain length (12C). XcDSF and BDSF are characterised by the presence of a double bond (*cis* 2), while XcDSF differs from BDSF by the presence of a methyl group at position 11. The chemical structure is drawn with Marvin JS by Chemaxon.

Figure S2.

Growth curve of *S. flexneri* with different concentrations of XcDSF and BDSF.

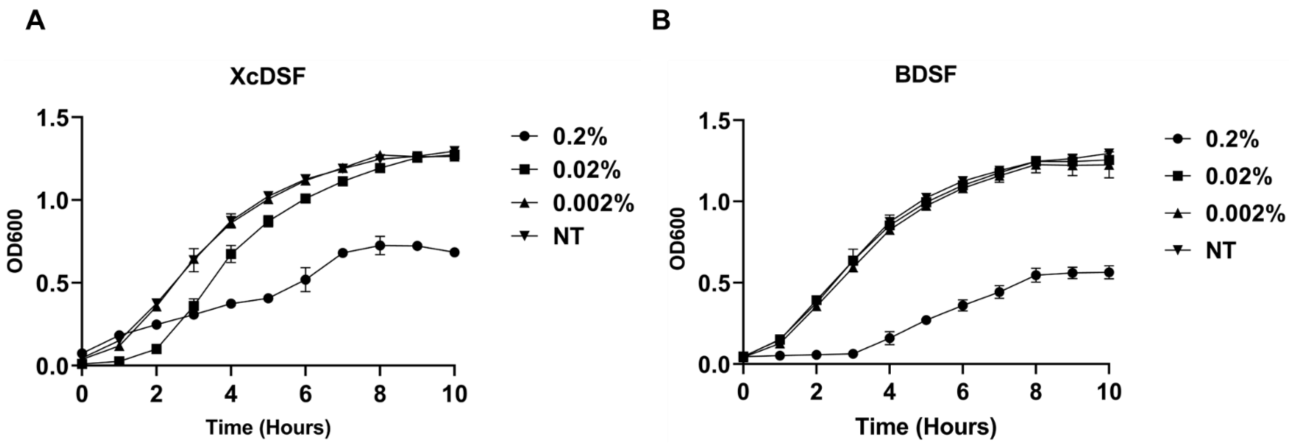

Figure S3.

Full size image of the western blots used to assemble fig. 2

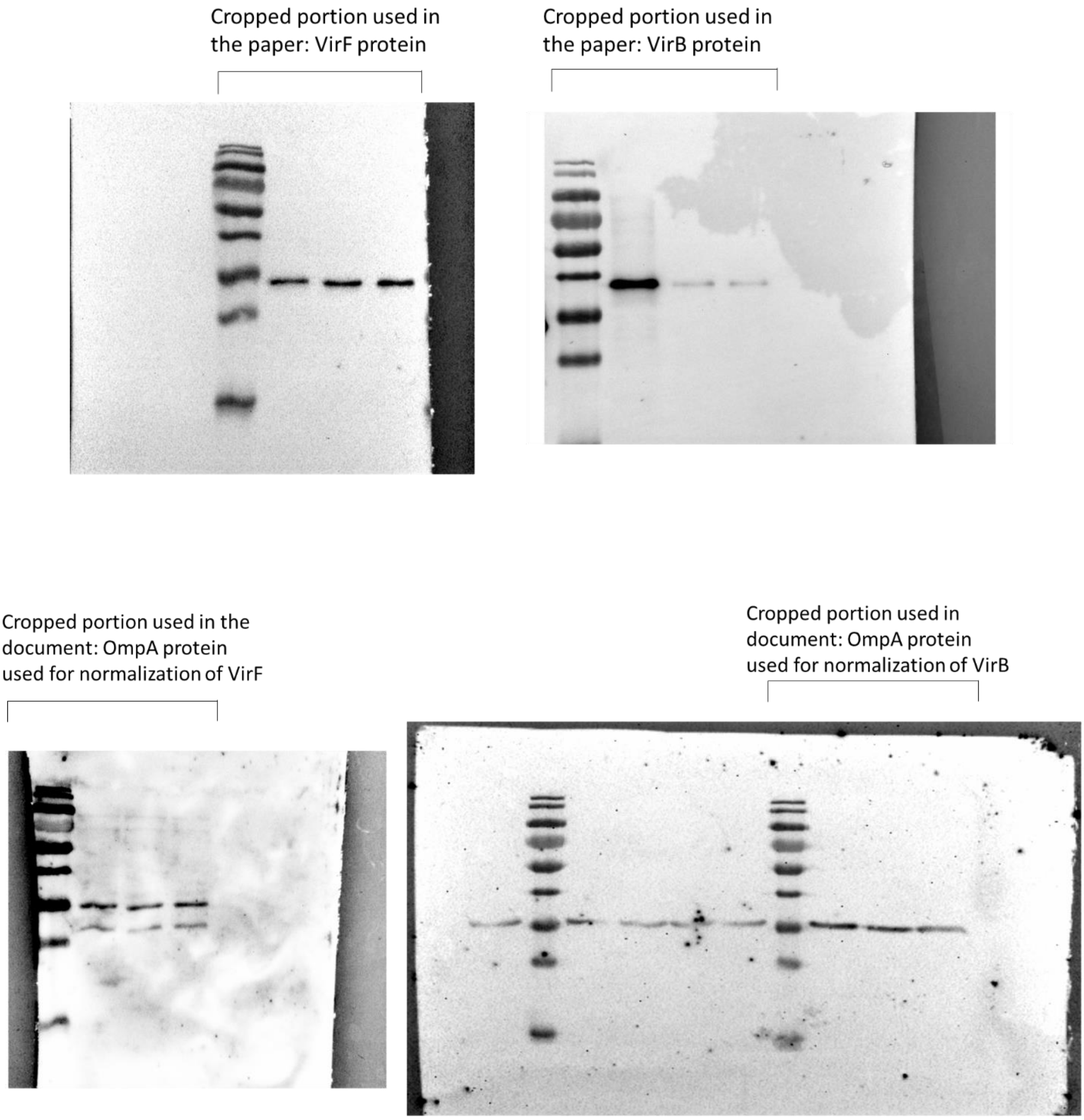

Supplement: Supplementary file 1 — Supplementary Information. [file 41598_2023_40023_MOESM1_ESM.pdf]
